# Epidermal Growth Factor Is Associated with Loss of Mucosae Sealing and Peri-Implant Mucositis: A Pilot Study

**DOI:** 10.3390/healthcare9101277

**Published:** 2021-09-27

**Authors:** José Jorge Schoichet, Carlos Fernando de Almeida Barros Mourão, Edgard de Mello Fonseca, Carlos Ramirez, Ricardo Villas-Boas, Juliana Prazeres, Valquiria Quinelato, Telma Regina Aguiar, Marina Prado, Angelo Cardarelli, Rafael Mello-Machado, Priscila Casado

**Affiliations:** 1 Graduate Program in Implant Dentistry, School of Dentistry, Universidade Federal Fluminense, Niteroi 24020-140, RJ, Brazil; jschoichet@terra.com.br (J.J.S.); edgardfonseca@vm.uff.br (E.d.M.F.); carlosramirez@globo.com (C.R.); ricovillas@gmail.com (R.V.-B.); julianaprazeres@gmail.com (J.P.); valquiriaquinelato@yahoo.com.br (V.Q.); telmaguiar1410@gmail.com (T.R.A.); marianaprado@gmail.com (M.P.); 2 Clinical Research Laboratory in Dentistry, Universidade Federal Fluminense, Niteroi 24020-140, RJ, Brazil; rafaelcoutinhodemello@yahoo.com.br; 3 Department of Dentistry, University Vita-Salute San Raffaele, 20100 Milan, Italy; angelo_cardarelli@libero.it; 4 Implant Dentistry Department, Universidade Iguaçu, Nova Iguaçu 26260-045, RJ, Brazil

**Keywords:** epidermal growth factor, mucositis, peri-implant tissue, mucosae sealing

## Abstract

This study aimed to evaluate the correlation between epidermal growth factor (EGF) and receptor (EGFR) levels in different clinical stages of dental implant rehabilitation and trace mucositis development’s biological profile. Thirty-six participants from the Specialization in Implant Dentistry, Universidade Federal Fluminense, Brazil, were included in the study and underwent sample collection: inside the alveolar socket, immediately before implant placement (Group 1, *n* = 10); at the peri-implant crevicular fluid (PICF) during reopening (Group 2, *n* = 10); PICF from healthy peri-implant in function (Group 3, *n* = 8); and PICF from mucositis sites (Group 4, *n* = 18). Quantitative polymerase chain reaction (PCR) evaluated EGF/EGFR gene expression using the SYBR Green Master Mix detection system. The results showed that EGF expression in the peri-implant crevicular fluid was statistically different. There was a higher EGF expression for group C (peri-implant health) (*p* = 0.04) than for the other groups. Regarding EGFR, there was no statistical difference among the groups (*p* = 0.56). It was concluded that low levels of EGF gene expression in the peri-implant crevicular fluid are related to the development of peri-implant mucositis and the absence of mucosae sealing. There was no correlation between EGFR gene expression with health or mucositis.

## 1. Introduction

Implant therapy has been widely applied in oral rehabilitation for many years, with predictable long-term results. The longevity and functionality of dental implants depend on the new bone formation around the implant body and the establishment of a soft tissue barrier called mucosal sealing that protects the underlying peri-implant structures and the implant itself. However, the presence of peri-implant mucosal inflammation (mucositis) can lead to loss of mucosal sealing and possible pathological bone resorption (peri-implantitis), culminating in implant loss [1].

In recent years, peri-implant diseases have become increasingly present in daily clinical practice. Despite differences in diagnosis, peri-implant mucositis is estimated to have a prevalence ranging from 50% to 80%, while peri-implantitis affects approximately 12–40% of implants [2], becoming a challenging problem for dentists [3]. In addition, many risk factors are associated with mucositis development, such as smoking habit, poor oral hygiene, history of periodontitis, alcohol use, long-term use of non-steroidal anti-inflammatory drugs, bisphosphonates, and uncontrolled diabetes Type 2 [3].

Peri-implant mucositis was defined by the Consensus of the 6th European Periodontics Workshop (WEP) as the presence of inflammation in the peri-implant mucosa without signs of loss of supporting bone [2,4]. Subsequently, the 7th WEP determined that the clinical parameter for the diagnosis of peri-implant mucositis is bleeding with soft force probing (<0.25 N) [5]. On the other hand, the American Academy of Periodontics (AAP 2013) [6] defined mucositis as the presence of bleeding on probing and/or suppuration, which is generally associated with clinical probing depth ≥ 4 mm, with no evidence of pathological radiographic bone loss. In 2019, the Consensus of the World Federation of Dentistry (FDI) characterized peri-implant mucositis by the presence of edema, bleeding on probing, redness, and/or purulent mucous secretion, further reaffirming its precursor function of peri-implantitis [3].

In this context, and with the increasing incidence of mucositis after implant placement, numerous studies have explored the biological characteristics of peri-implant mucositis that may be associated with the development or permanence of the disease. However, most of these studies focused on the exacerbated inflammatory response associated with the clinical pattern of mucositis, including analysis of interleukins, metalloproteinases, and different cytokines related to tissue destruction present in both peri-implant tissue and peri-implant crevicular fluid. As a result, most of these studies showed a high pattern of these pro-inflammatory markers associated with different patterns of tissue disruption around the implant [7,8,9].

However, studies of the characteristics of the peri-implant mucosa in health conditions, and its relationship with the formation of the mucosal seal, have shown peculiar characteristics present only in this tissue [1,10]. In addition to not having a periodontal ligament, which prevents its insertion into the implant, the peri-implant tissue has a pattern of less vascularization and more significant progression of tissue destruction when the mucosal sealing is disrupted, directly affecting bone tissue when compared to the periodontal tissue. Therefore, the presence of peri-implant mucosal sealing provides one of the only physical and biological barriers to the spread of peri-implant disease. This sealing, consisting of three epithelia (oral epithelium, peri-implant epithelium, and sulcular epithelium) is formed from the oral epithelium after the endosseous implant is installed [1].

Considering that, for the formation of the oral epithelium, the activity and expression of genes that enable tissue proliferation and stabilization of regenerative aspects is necessary, a study by Kim et al. (2012) [11] investigated, on a large scale, the possible regulatory molecules associated with the epithelial formation in the oral cavity, showing that the epidermal growth factor (EGF) is primordial and is highly related to the formation of the oral epithelium.

Epidermal Growth Factor (EGF) is a 53-amino acid polypeptide isolated initially from mouse salivary glands. The discovery of EGF was preceded by its ability to stimulate epithelial growth and differentiation after administration in newborn mice. The interaction of EGF with its receptor (EGFR) is known to trigger complex biochemical processes that lead to cell cycle progression. EGF is responsible for numerous primordial functions for wound healing in the oral mucosa since its functions include stimuli for cell proliferation, migration, and repopulation [11]. Low EGF levels in the peri-implant mucosa have been associated with the presence of marked mucosal inflammation, peri-implant mucositis, and loss of peri-implant mucosal sealing [12]. Nonetheless, countless questions remain unanswered: Is there a correlation between EGF and peri-implant mucosal sealing formation? Is there an association between EGF levels, loss of mucosal sealing, and development of peri-implant mucositis?

Molecular studies can help to understand the structural and biological properties of mucosal sealing and its interaction with a dental implant, showing mechanisms that orchestrate the integrity of the peri-implant soft tissue interface. However, data from human studies are still scarce [13]. Therefore, based on the biological function of EGF and the increased incidence of peri-implant mucositis, this study aimed to evaluate the correlation between the levels of epidermal growth factor (EGF) and its receptor (EGFR) at different stages. Clinically, it represents an evolution from the formation of mucosal sealing to the development of peri-implant mucositis through a cross-sectional study. We hypothesized a correlation between high EGF levels and the presence of peri-implant mucosal integrity and, in contrast, low EGF levels are associated with peri-implant mucositis. The null-hypothesis is that there is no correlation of EGF and EGFR level and mucosae sealing or mucositis. Deepening knowledge about the peri-implant mucosa composition may help in future therapies to maintain the biological seal or treat mucositis.

## 2. Materials and Methods

This research was conducted after the project submission and the respective In-formed Consent Form to the Research Ethics Committee (CEP) of the Hospital Universitário Antônio Pedro-School of Medicine, Universidade Federal Fluminense, having been approved by the number 2,455,991, in accordance with the provisions of Resolution 466/2012 and its complements to the National Health Council and Resolution 441/2011, addressing the inclusion of a biorepository.

All clinical and radiographic procedures were performed at the Clinic of the Specialization in Implant Dentistry, School of Dentistry, Universidade Federal Fluminense. Laboratory procedures were performed at the Clinical Research Unit.

### 2.1. Criteria for Inclusion and Exclusion of Research Participants

Inclusion Criteria: Healthy research participants were included; non-smokers; with and without previous history of periodontal disease, indicating installation of an endosseous implant (groups 1 and 2) or with already installed endosseous implants (groups 3 and 4), external hexagon; independent and lucid elderly, not dependent on other people/caregivers, and the informed consent form signed.

In groups 3 and 4, the research participants were required to have total fixed metal–plastic prostheses supported by mandibular screws installed for at least one year.

Exclusion Criteria: Report during anamnesis of systemic impairment (diabetes, blood dyscrasias), use of resorptive drugs and hormone replacement treatment, use of antibiotics, anti-inflammatory drugs, and mouthwash three months before the start of the study, and those considered vulnerable, according to Resolution 466/12.

In groups 3 and 4, research participants diagnosed with peri-implantitis and those who underwent peri-implant maintenance for at least 6 months were excluded.

A total of 36 participants were included in this study and divided into 4 groups, considering the bone healing period and load application: At dental implant installation (without loading) and assessment during the new bone formation: Groups 1 and 2. With load application on dental implants for at least 6 months: Groups 3 and 4.

### 2.2. Research Participants—Groups 1 and 2

A total of 10 participants were selected from the patients of the Universidade Federal Fluminense, Implant Dentistry Specialization course from March 2017 to April 2019. This number of participants was based on performing the sample calculation with 80% power.

These participants were all candidates for the installation of endosseous dental implants and were submitted to clinical/tomographic planning, according to the Implant Dentistry Specialization course protocol.

All participants were submitted to the installation of endosseous dental implant by the two-stage surgical technique, according to the protocol recommended by Misch et al. (2008) [14]. All implants placed were of the external hexagon with a regular platform (Implant: Titamax Ti Ex Cortical-Cylindrical-External Hexagon-Neodent^®^ (Straumann^®^ Group, JJGC Dental Materials Industry and Trade S.A.—Curitiba—Brazil), placed at the level of the alveolar bone crest.

All surgical procedures were performed by the same surgeon involved in the research (according to the instrumentation/milling protocol recommended by the manufacturer).

Clinical and radiographic analyzes were performed at two different times, as described below:

Group 1 (Immediate analysis after implant placement): clinical examination was performed after the dental implant placement, considering the primary stability and the immediate torque obtained. Digital periapical radiographs coupled to a radiographic positioner with an acrylic guide were used.

Group 2 (Bone healing analysis): it was performed with exposure of the implant 3 months after the installation surgery. Fifteen days after reopening, the implants were submitted to secondary stability measurement. Peri-implant tissue was clinically evaluated by mucosal staining and bleeding. The presence of implant mobility was also analyzed after the bone healing period.

### 2.3. Research Participants—Groups 3 and 4

All patients under treatment for peri-implant supportive therapy, in the period 2017–2019, at the Clinic of the Specialization in Implant Dentistry, School of Dentistry, Universidade Federal Fluminense, were considered possible volunteers to participate in the research.

Considering the inclusion and exclusion criteria, from a total of 42 patients, 26 participants were selected from these research groups, who after clinical evaluations were subdivided into 8 healthy participants (group 3—control) and 18 participants with peri-implant mucositis (group 4—mucositis).

### 2.4. Clinical and Radiographic Examination—Groups 3 and 4

A single researcher, calibrated through the kappa test, performed all the clinical steps of this study. The mandibular screwed implant-supported fixed total prostheses (hybrid denture) were removed, and all peri-implant implants and tissues were examined. Peri-implant clinical examination was performed on the mesial, distal, buccal, and lingual surfaces of each implant using a periodontal probe (model PCPUNC 156, North Carolina—USA, distributed by Hu-Friedy Brazil). Additionally, the distance from the edge of the pros-thesis to the mucosa was evaluated using a castroviejo dry-tip spectrometer (Golgran—São Caetano do Sul, Brazil).

The following clinical parameters were considered: peri-implant probing depth; bleeding on probing and/or suppuration; spontaneous bleeding, presence of mobility; the presence of plaque in the prosthesis and implants; keratinized tissue band; peri-implant biotype; mucosal color change; the presence of swollen area; exposure of implant threads; percussion sensitivity; and implant function time. The diagnosis of the regions analyzed was based on the parameters shown in Table 1.

According to the diagnosis, participants in this part of the research were divided into two groups: control group, characterized by peri-implant health in all implants, and mucositis group, characterized by the presence of peri-implant mucositis in at least 1 implant.

### 2.5. Considerations during the Clinical–Radiographic Examination in Research Groups

Radiographic exam: Radiographic examination consisted of digital periapical radiography using the Indicator Digital Shick Elite radiographic cone positioner—Indusbello (Londrina, Brazil). All radiographic shots were performed on the same X-ray machine DABI ATLANTE Spectro 70× (Ribeirão Preto, SP-Brazil) with the KODAK RVG5100 Digital Radiography System sensor (São José dos Campos, Brazil) and using the KODAK imaging program Software (São José dos Campos, Brazil), through a single operator. At the time of the radiographic examination, the participants wore a lead apron and a thyroid protector, complying with Federal Ordinance 453/98 (1 June 1998).

Physiological bone loss: physiological bone loss was characterized considering the normal bone loss of one millimeter during the first year after implant installation and 0.2 mm per subsequent year according to the bone healing period [2]. The bone loss calculation considered the diagnostic radiography and the protocol of implantation at the bone level for external hexagon implant. From this parameter, when the total bone loss (1 mm in the first year and 0.2 mm for subsequent years) was higher than expected, the diagnosis was peri-implantitis.

Patients diagnosed with peri-implantitis were referred for specific treatment at the Implant Dentistry Clinic.

All research participants with a diagnosis of peri-implant mucositis were referred for treatment in Peri-implant Supportive Therapy at the Implant Dentistry Specialization Course.

Diagnosis of periodontitis history was based on Berglundh et al.’s (2018) study [15] and it was performed before implant placement and considered radiographic aspects and clinical examination around the teeth, in all patients, as a clinical protocol.

### 2.6. Gene Expression Evaluation

**Figure 1:**
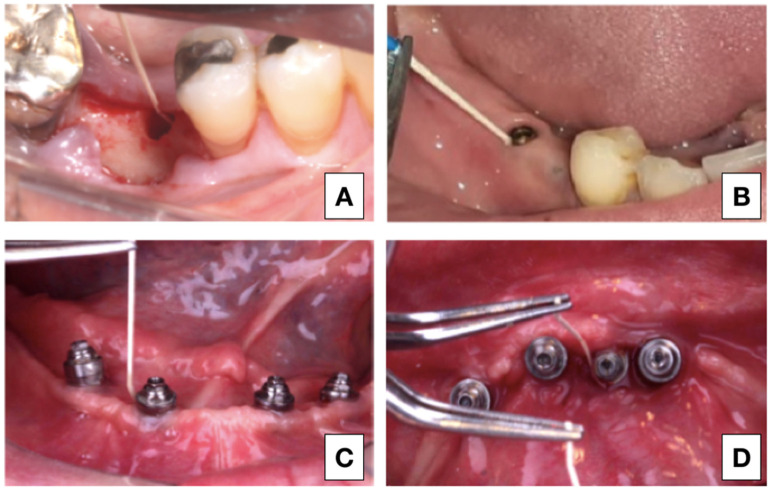
(**A**) GROUP 1 (*n* = 10): Sample collection from the surgical alveolus, previously implant placement. (**B**) GROUP 2 (*n* = 10): PICF collection 15 days after implant exposure. (**C**) GROUP 3 (*n* = 8): PICF collected from healthy peri-implant tissues after prostheses removal. (**D**) GROUP 4 (*n* = 18): samples from PICF in mucositis affected peri-implant tissues.

For the laboratory analysis, peri-implant crevicular fluid (PICF) was collected in the 4 different research phases. In Group 1, the collection was performed in the surgical socket before implant placement. For the groups submitted to the load, the place chosen for sample collection was the site with the highest degree of disease of each participant. In cases where all implants were healthy, the location of the implant closest to the midline was collected (Figure 1).

The mandibular screwed implant-supported fixed total fixed prosthesis (hybrid denture) was removed, and after relative isolation with a cotton roller, the implant surface in the collection area was dried. An absorbent paper filter (Maillefer—Dentsply, Petrópolis, Brazil) was inserted into the peri-implant groove for 60 s and immediately thereafter submerged in 1 mL TRIzol reagent and stored at −80 °C.

Total mRNA was extracted from the samples by the conventional TRIzol method (Invitrogen™ by Life Technologies, Waltham, MA, USA). DNase treatment to digest genomic DNA that could lead to false-positive results was performed using DNA-freeDNase^®^ (Ambion by Invitrogen™ by Life Technologies, Waltham, MA, USA). RNA integrity was confirmed and run on electrophoresis, agarose gel stained with 1.2% SYBR Stain^®^ (Invitrogen™ by Life Technologies, Waltham, MA, USA). RNA purity was confirmed by spectrophotometer absorbance ratio 260/280 and estimated RNA amount at 260 nm (Nanodrop^®^ 1000, Thermo-Scientific, Wilmington, NC, USA). The reverse transcription PCR (RT-PCR) reaction was performed for complementary DNA synthesis (cDNA) from 300 ng RNA using the ImProm-II Reverse Transcription System™ (Promega Corporation, Fitchburg, WI, USA) according to the manufacturer’s protocol. Blank control (RT-PCR without RNA matrix) and RT reactions (PCR reactions without reverse transcription) were performed together with all RT-PCRs. Quantitative PCR (qPCR) reactions were performed on the MxPro-Mx3005P software (Stratagene/Agilent Technologies, Wilmington, DE, USA) using the SYBR Green Master Mix (AppliedBiosystems, Foster City, CA, USA) with 1.5 µL of cDNA in each reaction. qPCR used activation at 95 °C for 10 min, followed by 40 cycles of denaturation and prolongation (95 °C for 15 s and 60 °C for 1 min). EGF specific initiators, EGFR were made based on BLAST data (http://blast.ddbj.nig.ac.jp/top-j.html accessed in 30 April 2019). The Livak method (2-ΔΔCT) determined the relative quantification of these gene expressions. The values were normalized to constitutive expression of β-actin. (forward 5′-AAT TAC GAG CTG CGT GTG G-3′ /reverse 5′-AGA GCG CAG GTA GGA TAG CA-3′).

### 2.7. Statistical Analysis

Numerical variables were expressed as mean ± standard deviation and subjected to the Normality Test (Shapiro–Wilk Test): normal (ANOVA e *t*-test) e non-normal (Mann–Whitney). Nominal variables were assessed by the chi-square test, including the odds ratio assessment with a 95% confidence interval. The Kruskal–Wallis test compared the 4 research groups simultaneously. The *p*-value < 0.05 was considered statistically significant. Gene expression analysis considered the normal distribution pattern, using the Mann Whitney test in the comparison between groups. Statistical analyzes were performed using the STATA11.1 software (StataCorp, College Station, TX, USA).

## 3. Results

### 3.1. Groups 1 and 2

Bone healing period: The 10 research participants had a mean age of 47.9 ± 8.2 years, with 5 (50%) women and 5 (50%) men, and only 1 (10%) participant was hypertensive. No participant had a history of periodontitis (0%).

Clinical results showed the absence of percussive and spontaneous pain, absence of peri-implant mucosal discoloration, and any other signs and symptoms of peri-implant inflammation and all had primary and secondary stability.

Considering the clinical and radiographic aspects, the regions studied at both intervals, before implant installation and after bone healing, showed no clinical signs of inflammation or peri-implant pathological bone loss.

### 3.2. Groups 3 and 4

Considering the inclusion and exclusion criteria previously described, from a total of 42 patients, 4 were excluded due to systemic impairment, 6 due to smoking, 3 due to a change of address (state), and 3 due to the diagnosis of peri-implantitis, totaling 26 participants.

**Figure 2:**
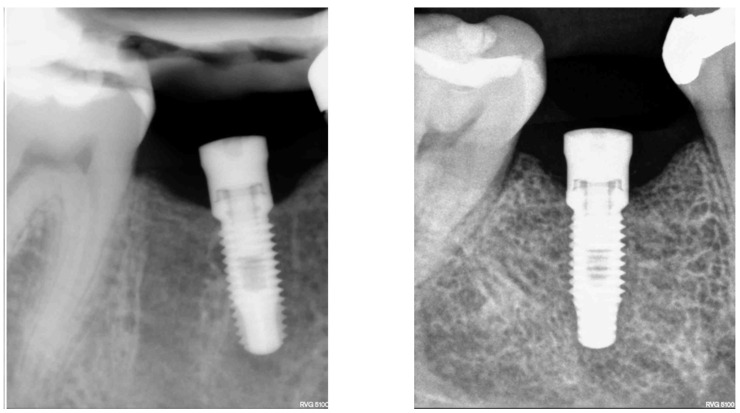
Radiographic aspect showing absence of peri-implant bone loss.

Considering gender, 16 (61.5%) participants were men and 10 (38.5%) women with a mean age of 65.5 ± 7.99 years. Based on the clinical parameters, no difference was found between the control and mucositis groups (Table 2). No radiographic bone loss was detected in any of the two groups (Figure 2).

### 3.3. Assessment of Group Homogeneity

The comparison between the clinical characteristics in the research groups showed that although the groups without load (1 and 2), groups 3 and 4 had different participants and were analyzed in different periods after implant installation, they did not present a statistically significant difference, considering age, gender, and history of periodontitis (*p* > 0.05), being considered similar for the statistical comparison relative to other parameters.

### 3.4. Gene Expression Results

**Figure 3:**
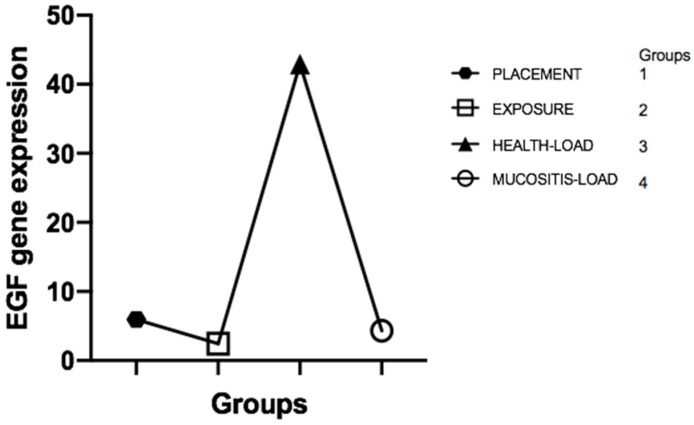
EGF gene expression in the groups, showing the high levels of EGF in Group 3 compared to the other groups.

The laboratory results showed, based on the Livak method calculation (2-ΔΔCT), that the expression of EGF mRNA in the peri-implant crevicular fluid (PICF) was significantly higher in group 3 (peri-implant health) compared to the other groups (*p* = 0.04) (Figure 2 and Table 3). The Kruskal–Wallis test confirmed the results, showing a statistically significant difference between the groups when compared together (*p* = 0.04). Considering EGFR gene expression levels, the results showed no statistically significant differences among the groups (*p* = 0.56) (Figure 3).

**Figure 4:**
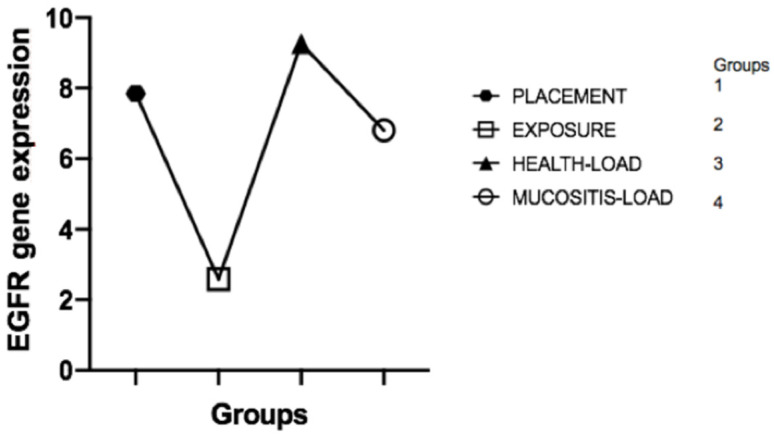
EGFR gene expression in groups 1, 2, 3, and 4, showing no statistical difference.

**Figure 5:**
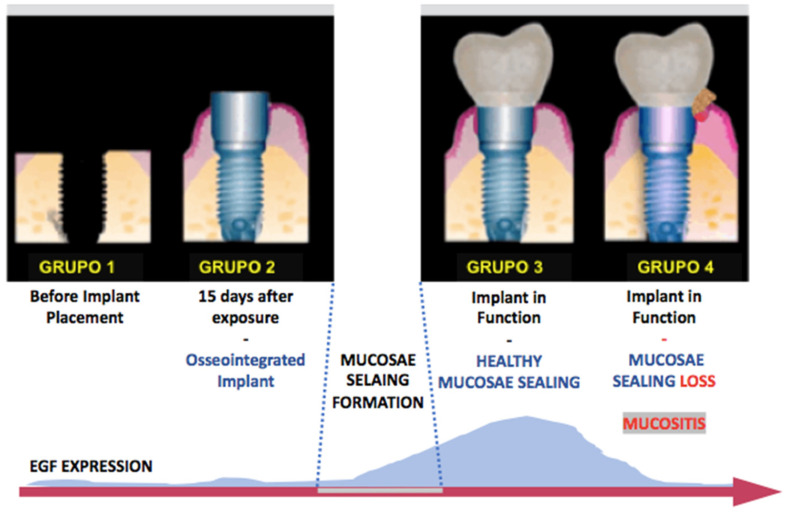
Illustrative representation of the correlation between mucosal sealing and peri-implant health and high levels of EGF.

According to clinical results and EGF expression, it can be observed that the increase in EGF is compatible with the presence of mucosal sealing, and its decrease in implant function is associated with the presence of mucositis (Figure 4 and Figure 5).

## 4. Discussion

Peri-implant mucositis is clinically characterized by signs compatible with an inflammatory response in the mucosa surrounding the implant. The rates of this disease have been growing significantly in recent years, stimulating studies looking for its etiology and main treatments. One consequence of mucositis is the loss of peri-implant mucosal architecture and integrity, suggesting an instability associated with growth factors responsible for tissue maintenance around implants. However, there is little research on the function or formation of soft tissue sealing around dental implants, and the biological characterization of this interface remains unclear [1]. Thus, this cross-sectional study aimed to evaluate the correlation between the levels of epidermal growth factor (EGF) and its receptor (EGFR) in the different clinical phases that represent an evolution from the formation of mucosal sealing to the development of peri-implant mucositis. We believe that this broad assessment of EGF and EGFR may partly explain the correlation between maintaining biological sealing and the presence of long-term peri-implant health. In fact, our main results showed that there is no difference between EGF and EGFR levels in the crevicular fluid before and after implant placement (bone healing period) where loading was not performed; after loading the implant, the healthy and healthy mucosal sealing clinically compatible with peri-implant health presented significantly higher EGF expression than the unloaded or mucosal tissues with clinical presence of peri-implant mucositis, characterized by loss of mucosal architecture, and is associated with a significant decrease in EGF levels in the implants in function; EGF levels, in the presence of peri-implant mucositis, returned to levels similar to those found around the implants after the bone healing period, in which they were not functioning and did not have the mucosal seal formed; there is a clinical correlation between the peri-implant health of implants in function and elevated EGF levels; elevated EGF levels are associated with the presence of intact mucosal sealing which is against our null-hypothesis, showing that low EGF level after function can be associated with clinic mucositis.

Peri-implant mucositis is mainly caused by the formation of a bacterial biofilm, which triggers an exacerbated local inflammatory response, leading to the destruction of peri-implant soft tissues [2,15]. There is sufficient evidence to conclude that several general risk factors impact the short- and long-term success of implant therapy.

Just as prevention and treatment of peri-implant mucositis are related to long-term success in implantology, mucositis is the obvious precursor of peri-implantitis, a significant cause of implant loss [5]. Therefore, this study used strict criteria for the inclusion of research participants to minimize possible factors that could confuse the etiology of this multifactorial disease (i.e., excluding smokers, patients with systemic disease, using resorptive drug therapy, and hormone replacement treatment), thus making the biological results even more significant. Another factor that we consider even more relevant is the fact that the groups involved in this research were homogeneous, without a statistical difference, regarding the general clinical aspects, such as age, gender, and history of periodontitis, which further emphasizes the results.

Another critical issue in the study of peri-implant diseases is the representativeness of peri-implant crevicular fluid, which has been characterized as a promising means for detecting peri-implant activity [16]. Biochemical mediators secreted in the PICF were considered diagnostic markers to monitor peri-implant health [17], reflecting the degree of inflammatory and regenerative reaction that affects surrounding tissues, bones, and mucosa [18]. Our study considered collecting PICF, not only because it represents metabolism at the tissue-implant interface, but because it is a non-invasive method that can be applied in future clinical practice for future early diagnostic or therapeutic approaches. Our laboratory results have shown that PICF is a safe and predictable source of peri-implantable and processable tissue-associated cells for analysis of cellular expression through mRNA.

The mucosa around the implants forms a seal that is similar to the junctional epithelium in the periodontium. This peri-implant union is composed of three types of epithelium: peri-implant epithelium, peri-implant sulcular epithelium, and oral epithelium [19,20]. The peri-implant epithelium performs an epithelial bond with a similar function to the junctional epithelium and forms from the oral epithelium within two to three weeks after implant placement. Morphologically, the peri-implant epithelium is composed of a thin layer of three–four cells and has immunoglobulins, neutrophils, lymphocytes, and plasma cells in a wide intercellular space, which together [1] protect the underlying tissue from deleterious exogenous factors. However, the peri-implant epithelium has a lower functional sealing capacity when compared to the junctional epithelium, despite having similar epithelial structures [10]. This fact influences the preservation of the mucosal seal, adding that the oral epithelium, a component of the peri-implant mucosa, has low adhesion to titanium, probably caused by the electrostatic characteristics of the implant and the elution of ions [1]. On the other hand, the underlying peri-implant connective tissue is characterized by the presence of type V collagen fibers, which have no insertion to the implant, making this tissue a chronic inflammatory condition, not an interception or defense structure. In addition, fiber orientation and patterns of attachment of the epithelium to the implant and tooth are fundamentally different due to the absence of cementum and periodontal ligament around the implant [19].

Considering the natural biology of peri-implant mucosal seal formation and considering that the integrity of this seal is associated with the maintenance of peri-implant health, as it is the main barrier to external insults, our study investigated the expression of markers related to mucosal regeneration before and after the formation of this seal, culminating in the health transition to peri-implant mucositis, characterized by the loss of the seal. The mRNA expressed by cells in the FCPI showed that EGF and EGFR gene expression during implant installation and two weeks after dental implant exposure surgery showed no statistically significant difference. This result is closely related to the fact that the mucosal sealing was not completely formed during these two ICF collection periods. Its formation begins from the oral epithelium two–three weeks after the implant contact with the oral epithelium [1]. In our study, this contact occurred after the bone heling period, with the PICF analysis being performed prior to the establishment of this seal.

However, it became evident that healthy peri-implant regions in implants subjected to loading showed a significant increase in EGF gene expression. This fact may be based on numerous justifications. First, healthy peri-implant tissues are characterized by the presence of an intact mucosal seal that possibly retains its architecture and function due to the activity of growth factors such as EGF, closely associated with epithelial regenerative capacity. Another explanation, which we consider plausible, is that the presence of load on the implant may cause tissue microtrauma that needs a constant regenerative response to maintain mucosal integrity, different from what occurs in the newly unloaded implant, thus presenting low EGF levels compared to healthy tissues around prosthetic-connected implants. Perhaps there is a positive regulation between mechanical implant functionality, mucosal seal formation, and high EGF levels.

On the contrary, we observe that when peri-implant tissue loses mucosal sealing in implants subjected to loading due to the presence of exacerbated mucosal inflammation characteristic of peri-implant mucositis, EGF levels fall dramatically in the PICF. This fact may represent an influence of the destructive tissue inflammatory response in the peri-implant, culminating in the destruction of cells capable of producing EGF and maintaining tissue integrity, thus activating a cascade effect of mucosal sealing regenerative incapacity coupled with the high inflammatory response. At this point, the indication of some therapeutic techniques is justified in order to prevent the development of peri-implantitis and implant loss. Del Amo et al. (2016) [21] and Renvert et al. (2019) [22] emphasized the removal of the implant-supported prosthesis and the mechanical and chemical treatment of the peri-implant mucosa as a way to reverse the pathological condition of mucositis. In fact, biologically, based on our results, the absence of implant loading decreases tissue trauma and, along with the removal of other etiological factors, such as biofilm accumulation, can dramatically decrease peri-implant cell death, thus increasing the tissue regenerative capacity. Thus, the elevation of EGF levels after mucositis treatment may return to clinical signs compatible with health. However, future research is needed.

EGF’s biological activities depend on its binding to a specific cell membrane receptor, through which it exerts a potent mitogenic effect on most epithelial cells, fibroblasts, and endothelial cells [23]. EGF-receptor interaction triggers complex biochemical processes that eventually lead to cell cycle progression [24]. In patients with diabetes, decreased EGF levels are primarily responsible for impairing fibroblast functionality, limiting extracellular matrix formation, and decreasing angiogenic response [25]. EGF has been shown to be responsible for numerous primordial functions for wound healing in the oral mucosa since its functions include stimuli for cell proliferation, migration, and repopulation [11]. Atsuda et al. (2016) [1] suggested that adhesion by hemidesmosomes to the implant surface is produced by epithelial cells from the implant surface.

Other interesting finds were from Óbice et al. (2019) [26]. The authors suggested that osteoblastic activities and angiogram are predominantly observed in the early stages of peri-implant regeneration, considering high levels of EGF, among other markers, in the peri-implant fluid during this period. It should be noted that these osteoblastic and angiogenic activities were observed in the initial phase (30 days postoperatively), which may suggest an acceleration in the process of bone neoformation around the implants/components and, consequently, a biological sealing—fastest mucous membrane of this interface.

Based on the regenerative potential of EGF, numerous therapies have been suggested and tested for wound healing and increased tissue regeneration. EGF is one of the most significant growth factors in therapies involving Platelet Rich Plasma for bone regeneration, being applied in the dental clinic for different purposes. In 2019, Pansani et al. [27] evaluated the potential of EGF-coated titanium surfaces to increase the adhesion potential of oral mucosa cells to simulate peri-implant mucosal seal formation. The results were promising, showing that EGF was able to stimulate the adhesion and metabolism of gingival fibroblasts, being an alternative for the maintenance of mucosal sealing.

However, so far, few studies have investigated the peri-implant adhesion structures and their biology, which is necessary to maintain peri-implant health and implant longevity. This study is the first to describe differences in EGF expression before and after mucosal seal formation and its association with the presence of peri-implant mucositis. Some limitations found were the impossibility of characterizing peri-implant mucosal sealing, considering that the work was performed in humans, the lack of information on EGF levels after treatment of peri-implant mucositis, and the number of participants in the research. Other limitations include the follow-up of the same participant from implant placement (group 1) to the possible development of mucositis (group4). However, it would limit the number of samples and make the study execution time unpredictable. Maybe this model can be reproduced in animal studies.

Dental research in the field of genetics has been introduced in universities as a vital part of developing a thriving and strong educational system in its three pillars—Education, Research, and Extension—and studies are growing as new faculties of dentistry incorporate and mature a solid research profile in their educational programs [28], opening up a diagnostic possibility and possible future therapeutic proposals.

From the results obtained in our work, as well as the clinical experience of the present day, the success of implant-supported prosthetic rehabilitation has become more straightforward and predictable. However, there is still a range of individuals for whom the prediction is not so simple. The patient’s susceptibility to developing exacerbated pro-inflammatory reactions may, in part, be an etiological factor in the failure of dental implants and surrounding soft tissues. Specifically, in these cases and perhaps in general, if possible, EGF and EGF-R levels may contribute to the optimization of mucosal seal results around implants and prosthetic components. Future human clinical studies, including these, among other molecules, may play a key role in their influence on regenerative processes and peri-implant health.

## 5. Conclusions

Based on our results, the low levels of EGF gene expression in the peri-implant crevicular fluid are related to the development of peri-implant mucositis and the absence of mucosae sealing. Futures therapies based on EGF clinical application in mucositis treatment should be investigated in futures researches. There was no correlation between EGFR gene expression and health or mucositis.

## Figures and Tables

**Table 1:** Diagnosis of the peri-implant diseases studied (based on LINDHE & MEYLE, 2008) [2].

Peri-Implant Health (Group 3)	Peri-Implant Mucositis (Group 4)
Absence of: Spontaneous Bleeding Probing Bleeding Edema Color Change Purulent Secretion Pathological Radiographic Bone Loss	Presence of: Bleeding On Probing Tissue Color Change Peri-Implant Edema Purulent Secretion Absence of pathological radiographic bone loss

**Table 2:** General characteristics of research participants and clinical aspects in groups 3 and 4.

Parameters	Control *n* = 8 (*n*/%)	Mucositis *n* = 18 (*n*/%)	*p*-Value (OR; IC) *
Gender			0.10 (0.23; 0.03–1.34)
Masculine	3 (37.5)	13 (72.2)	
Feminine	5 (62.5)	5 (27.8)	
Age	67.75 ± 2.1	64.72 ± 2.0	0.38
Periodontitis History	5 (62.5)	8 (44.4)	0.33 (2.08; 0.37–11.48)
Gender			0.10 (0.23; 0.03–1.34)
Masculine	3 (37.5)	13 (72.2)	
Feminine	5 (62.5)	5 (27.8)	
Clinical Aspects			
Implant in function (years)	4.18 ± 4.15	3.38 ± 1.42	0.99
Total number of implants	4.25 ± 0.46	4.55 ± 0.70	0.26
Plaque buildup in the prosthesis	8 (100)	13 (72.2)	0.15
Antagonist			
Teeth	0	3 (16.7)	0.33
Dentures	7 (87.5)	11 (61.1)	
Implant-supported prosthesis	1 (12.5)	4 (22.2)	
Prosthesis-mucosa distance (mm)	2.56 ± 5.05	0.72 ± 0.77	0.35
Peri-implant Biotype			0.17 (0.33; 0.06–1.69)
Thin	5 (62.5)	10 (55.6)	
Thick	3 (37.5)	8 (44.5)	
Peri-implant plaque	8 (100)	17 (94.4)	0.31
PCS **	2.37 ± 0.51	2.61 ± 0.97	0.60
Keratinized mucous thickness	2.62 ± 1.18	1.61 ± 1.33	0.07

* OR: odds ratio; CI: confidence interval; ** PCS: clinical probing depth.

**Table 3:** The *p*-values in the comparison between the groups, considering the EGF gene expression.

	Group 2 Exposure	Group 3 Peri-Implant Health	Group 4 Peri-Implant Mucositis
Group 1 * Implant Placement	0.75	0.01	0.70
Group 2 Exposure	NA	0.01	0.89
Group 3 Peri-implant Health	NA	NA	NA
Group 4 Peri-implant Mucositis	NA	0.02	NA

* Group 1 was the reference for comparison among groups. (NA = Not available).

## Data Availability

The data presented in this study are available on request from the corresponding author.
